# Negotiating the Joint Career: Couples Adapting to Alzheimer's and Aging in Place

**DOI:** 10.1155/2012/797023

**Published:** 2011-12-18

**Authors:** Renée L. Beard, Sasha Sakhtah, Vanessa Imse, James E. Galvin

**Affiliations:** ^1^Department of Sociology and Anthropology, College of the Holy Cross, One College Street, Worcester, MA 01610, USA; ^2^Departments of Neurology and Psychiatry, Center of Excellence on Brain Aging, NYU Langone Medical Center, New York, NY 10016, USA

## Abstract

To understand the impact of memory loss on aging in place, this paper investigated dyads where one spouse had been diagnosed with memory loss. In-depth qualitative interviews were conducted with ten couples (*N* = 20). Grounded theory methods were used to collect, code, and analyze data into themes. Data revealed consensus among and between dyads that it was best to focus on living, rather than what had been or might someday be lost. Nonetheless, differences according to gender and cognitive status (e.g., diagnosed or spouse) were reported. Given population aging, identifying the impact of gender roles and social norms on the potential for aging in place with memory loss is critical. Community services and care practices must be sensitive to the ways that couples prioritized and organized their relationship prior to diagnosis in order to encourage positive patterns of care between couples, foster successful adaptation to changing needs, and support in-home arrangements as long as possible.

## 1. Introduction/Background

Recent policy and service initiatives have focused on helping older adults remain in their current homes for as long as possible, that is, to “age in place.” Research has shown that older adults prefer to stay in their homes [1]. In addition, movement from familiar surroundings can have deleterious effects on health, economic factors, and quality of life, especially for persons with dementia [2–4]. A recent report [5] explored anticipation of relocation from a naturally occurring retirement community (NORC) in a nonrandom sample of 324 community-dwelling older adults. The 26% of NORC residents who worried they would have to move cited physical health, finances, physical structure of the home, and social isolation as the most common reasons. 

Over the past few decades, a rich literature on aging in place has flourished within environmental gerontology. The interdisciplinary field has examined the unique role of place, or the built environment, on experiences and meaning of life. Environmental factors are now recognized as having a significant impact on quality of life for seniors in general [6–9] as well as persons with dementia in particular [10–15]. Scholars have explored the meaning of home to seniors and how this may be fostered or disrupted by changes related to aging [7]. Although the vast majority of this work focuses on institutional settings, quality of experiences in long-term care that is not nursing home care has been examined [8–10]. This is especially important for seniors living alone since the home environment and personal space are often the most significant variables facilitating their independence [8]. Home takes on a particular meaning in old age because it can compensate for reductions in functional and/or cognitive abilities [9].

The importance of physical and social environments for ensuring quality dementia care has been of growing interest to the field [12]. Research [14] suggests that built environments are shaped by unifying philosophies or goals and that “relationships between the various components of place—organizational, social, architectural, and experiential—are typically far from random,” but instead “work towards the overarching goal of the environment shaping social attitudes and patterns of behavior” [15, page 13]. This model argues that built environments are experienced through perception, cognition, action, affect, and meaning. “For people with dementia,” others add, “memories of the places in their lives and the events, emotions, and experiences associated with those places may help provide continuity even as cognitive and communicative abilities dwindle” [11, page 8]. Accordingly, evoking such memories can assist in “rediscovering the self” for persons with dementia. 

The role that family members can play in eliciting the shared biographies (and events that predate the family member) is crucial to such self-preservation. Although this work explicitly addresses reminiscence as a tool to be used in institutional settings, many of the recommendations can be extended to the individual home environment as well. Since “place and memories of place play an important role in shaping—and sustaining—our sense of self” [11, page 22] and memories of home are part of our self-identity, family members of persons with memory difficulties can prompt pleasant past experiences. This model is in line with Kitwood's seminal thesis that “person-centred care” involves knowing the life history of individuals with dementia [16]. In this case, compassionate others can support the narrative identity of persons struggling to remember. 

This impressive body of research elucidates the critical role family members, particularly spouses, can play in helping individuals with memory loss reminisce, and thus maintain self-identity. It also highlights the unique features of aging in place after having lived somewhere for several decades as a couple. For example, persons who lived at a home prior to becoming forgetful will arguably be able to maintain more independence in a place that is familiar and involves routines than they might be if relocated. Environmental gerontologists have discussed the ability of personal experiences to attribute meaning to places in terms of “insideness” [6]. For persons living with dementia who reside with a spouse, experiential familiarity or “physical insideness,” patterns of interdependence or “social insideness,” and a sense of belonging or “autobiographical insideness” are all crucial to aging in place. Both the existence of familiar routines and reminders of significant life events and places can help provide continuity in spite of cognitive decline, thus allowing persons with dementia to potentially stay at home far longer than might be the case otherwise.

### 1.1. Alzheimer's Disease

With the aging of the populace, individuals are more likely to develop chronic diseases such as Alzheimer's (AD). Historically, research has largely overlooked the social interactions and sociocultural frameworks in which forgetfulness occurs [17, 18] despite the fact that psychosocial factors influence the quality of aging experiences. The majority of older adults have multiple chronic medical conditions that may impair everyday function leading to increasing vulnerability [19]. These conditions may affect the ability to make independent decisions, forcing older adults to rely more heavily on outside forces such as their spouses for assistance in making major decisions regarding health, lifestyle, and living arrangements. Knowledge of proximal determinants of behavior is important because it allows identification of variables that are more readily amenable to change [20].

Alzheimer's disease and its prodrome, mild cognitive impairment (MCI), have historically been interpreted within a biomedical framework, and the assumed impact has been presented as universally devastating for diagnosed individuals and family members alike. Cross-disciplinary research demonstrates that social perceptions of dementia are generally negative [21–23], yet subjective experiences of living with the diagnosis and providing care vary by racial/ethnic, socioeconomic, and gender status [24, 25]. While many studies have focused on the individuals' struggle to avoid assuming the (pejorative) Alzheimer's identity [26–29] or the courtesy stigma of having a family member with dementia [30, 31], only recently has research begun examining dementia as experienced by couples [32–37]. In addition to the trend of increasingly earlier diagnosis, this shift has likely been precipitated by the nearly 5.3 million Americans with AD being cared for within their homes, primarily by spouses [38]. 

Previous studies on “couplehood” suggest that the experiences of persons with AD and their spouses are heavily influenced by a medicalized explanatory framework, the reciprocity of spousal bonds, and stereotypical rhetoric of loss [35, 36]. Alzheimer's diagnoses create an opportunity for both diagnosed individuals and their family members to either accept or reject the label. In support of previous research [26, 27], individuals often vacillate between openly embracing the label and trying to avoid being associated with its “master status,” therefore both minimizing and exploiting processes of medicalization. This simultaneous acknowledgment of and resistance to the label has been used to highlight the inappropriateness of talking about the “acceptance” or “denial” of AD [39]. Understanding the Alzheimer illness identity as pendular rather than linear is, therefore, particularly salient. Previous findings have suggested that couples work together, describe dementia as part of the normal aging process and focus on positive thinking to avoid the social stigma associated with an Alzheimer's diagnosis [35] and to maintain both personhood and couplehood. 

Debates over the relationship between illness and identity [40, 41] represent diagnosed individuals' struggle between biomedicine and a sense of self-worth [42]. Using a symbolic interactionist and social constructionist lens [43–45], we examine how individuals diagnosed with memory loss and their spouses make sense of and manage the deeply emotional and stigmatizing experience of living with memory loss. A couple's interpretation of AD is affected not only by a medicalized society, but by their unique social and relational contexts. 

Research demonstrates that negotiations between AD and the self are dependent on spousal interactions, with support persons potentially providing extensive restorative identity work [46] to downplay (the exclusively negative) AD identity and to maintain couplehood. Using Glaser and Strauss' seminal work on awareness context theory [47], we argue that experiences of memory loss are based on relational capacity that will be negotiated differently by a couple than an individual and impacted by factors unique to each couple. Furthermore, during “shared awareness” both the diagnosed individual and the care partner will remain active [35]. Through investigating dyadic relationships (predominantly defined as couples but other caregiver-recipient pairs have also been utilized), studies have begun to demonstrate a sense of “we do it together” and a breaking away from the traditional pejorative framings of Alzheimer's disease and so-called “caregiving.” An innovative body of research has reported noteworthy exceptions to this assumption by demonstrating positive and/or spiritual experiences of both living and caring for someone with Alzheimer's [26, 28, 48–51]. Recent studies have even suggested that enhancing the dyadic relationship may help slow Alzheimer's-related decline [52] and decrease psychosocial costs to carers [53]; thus encouraging positive adaptation to the condition and increasing the likelihood of aging in place. Indeed, even interventions aimed at improving social support and coping skills have effects on both parties, including reduced caregiver depression, improved well-being, and delayed entrance into nursing homes [54, 55]. It is now well documented that diagnosed individuals understand the social and psychological aspects of AD, including social (mis)perceptions of diminished dignity and value [56]. Accordingly, for those who are coupled, this crucial social relation can be essential to positive experiences of memory loss and continued in-home living. This is the basis for our analysis, as we found evidence in stark contrast to the traditionally negative view of “suffering” in relation to memory loss. This article will explore the role of spousal dyads on efforts to adapt to memory-related changes and to age in place. 

This study compares how diagnosed individuals and those who care for them define AD and narrate their subsequent experiences and how this impacts efforts to maintain personhood, couplehood, and the desire and/or ability to remain living at home together. Based on recent findings that dementia is a “collaborative venture” and that the “us identity” of the couple is preserved in spite of the diagnosis of dementia [33], we, too, investigate couples' joint production of meaning. We also add to the research on “couples' shared constructions of, and responses to, the diagnosis” that reveals oscillating processes of meaning-making and adjusting to dementia [37, page 337]. Thus, we borrow from Karp [57], when we argue that spouses have a “joint career” of memory loss; a career which is socially constructed and negotiated. In so doing, our data suggest that dyads dealing with memory loss together, or “negotiating its impact” [58] and “co-constructing caring” [59], are more likely to remain positive, maintain a sense of self and couplehood, remain living at home, and evade the typically negative framework attached to the condition in America. 

## 2. Methods

These data are a subset of a larger study, entitled ACCESS (Assessing the Cultural Characteristics of Elders and the Support Systems), exploring first- and second-hand narratives of early-stage memory loss. The purpose of the research was to examine everyday life with memory loss, including AD and MCI. The sample reported here contained only dyad interviews and is analyzed separately from the larger cohort to permit investigation of the impact of gender and relationship to memory loss (diagnosed or support person) on the joint experiences of couples confronting AD/MCI. Positing that couples interact within marriages by constructing symbolic and shared realities, we sought to investigate how their common perceptions might be altered by memory loss. That is, how do couples make sense of and respond to memory loss and the impact it has on their relationship? If meaning is produced as a joint venture within dyads, then how might memory loss influence their shared reality?

Ethical approval for the study was obtained from the Institutional Review Board at the University of Illinois Chicago (Protocol no. 2005-0839). The present paper reports on in-person dyadic interviews conducted with coresident, married couples dealing with memory loss. All interviews took place in the couples' home. The research was based on a nonprobability sample using convenience sampling. Grounded theory techniques were employed to collect, code, and analyze data by consolidating textual data into broad themes.

### 2.1. Sample

The inclusion criteria for participation were expert diagnosis with AD/MCI at a specialty memory clinic and the presence of a spouse dyad. These criteria allowed us to target the experiences of those diagnosed with memory loss and their family members. The sample included ten dyad interviews with individuals experiencing memory loss and their spouses (*N* = 20). All ten individuals diagnosed with memory loss were evaluated at a specialty memory clinic and meet criteria for either Alzheimer's or mild cognitive impairment. Criteria included MMSE (Mini-mental state examination (MMSE; Folstein, Folstein and McHugh 1975) scores range from 0–30, with 30 being perfect.) scores between 20–30 (out of a possible 30), where lower scores signify the presence of progressive cognitive impairment. The average MMSE score for diagnosed respondents was 25 (ranging from 22 to 30). Individuals diagnosed with MCI did not meet clinical criteria for AD, but had subjective memory complaints and were greater than 1.5 standard deviations below the norm (adjusted for age and education) on the neuropsychological tests. All ten diagnosed individuals were considered in the early stages of AD (*n* = 7) or its prodromal stage MCI (*n* = 3; at the time this research was conducted, a clinical diagnosis of MCI was considered a potential precursor to Alzheimer's. New diagnostic guidelines released jointly by the US Alzheimer's Association and National Institute on Aging in April, 2011 redefined MCI as an official “stage” of Alzheimer's). The average time since diagnosis was just under 3 years, but close to half had been diagnosed roughly two years prior to being interviewed. 

Our dyads included 6 male patient-female spouses and 4 female patient-male spouses. Ninety percent of our couples lived in urban settings, with only one dyad residing in a rural area. The vast majority of the respondents stated their race as Caucasian, with one couple identifying as Hispanic. All dyads lived together, and one couple had young children living in their household as well. All informants completed at least twelve years of schooling; 9 of the 10 couples having at least some college experience. The mean age of our respondents was 73 (ranging from 50 to 89 years old). The median income was $65,000–$99,999, with only one couple reporting an income level below $20,000. No significant differences in demographic characteristics between the male diagnosed-female spouse and female diagnosed-male spouse dyads or the AD and MCI dyads were found. See Table 1 for the specific demographic data on the complete sample.

### 2.2. Qualitative Analysis

This research was informed by an inductive method of data collection, sampling, and analysis. Rather than strictly testing hypotheses or applying existing theories to data, the aim was to generate theory that is “grounded” in the data itself. As such, the product of this research is a midrange substantive theory and its generalizability lies in the concepts discovered within the sample studied rather than the larger population from which it was drawn. 

The first author, and principal investigator, conducted all interviews in person using a semistructured interview guide (see Table 2). Since respondents were seen as the experts on the topic, interviews were conducted in an informal, open-ended manner to allow dyads to tell their unique stories of memory loss. Given the dearth of existing data including both perspectives simultaneously, interviews aimed to elicit the shared story of each couple rather than test specific hypotheses. The questions from the interview guide were used as probes to generate conversation without following a standardized format. The only question that was asked of every dyad was: “Can you tell me what your life together has been like since [name] was diagnosed with memory loss?”

All interviews were audio-taped, transcribed, and then analyzed using the constant comparative method and coding paradigm of grounded theory [60]. Since this method aims to consolidate information into matrices in an effort to generate overarching themes, the on-going process of taking notes, writing memos, and (re)reading data lends itself to emergent categories for simplifying and articulating data. 

Detailed notes were dictated immediately following all interviews. Paid assistants transcribed each taped interview verbatim, yielding 311 pages of narrative transcript data. The PI verified transcript accuracy by reading each one upon receipt. Additional quality control measures (i.e., listening to transcripts while playing the data files) were performed on 100% of the subsample used for the present analysis. 

Analysis began with “open coding,” which involved identification of the dimensions and properties of the themes in the margins of textual data line by line. Next, themes were consolidated by using an explanatory matrix to identify major “core variables.” To ensure reliability of the findings, two research assistants (SS and VI) read all textual interview files, provided detailed analysis notes, and line-by-line coded each transcript. The common themes for the subset of spousal dyads will be reported here.

## 3. Findings

In support of previous research [32, 35], couples in our study did not dwell on their respective experiences of AD. Rather, they expressed a desire to “keep on keeping on,” which appeared to minimize the impact of a diseased identity and help avoid their being consumed by the condition. As reported elsewhere [37, 61], this led them to try to emphasize the “retained abilities” [29] of the diagnosed counterpart and their joint identity rather than focus primarily on the various losses, or symptoms, associated with memory loss. Importantly, this involved both parties knowing when they needed support. 

### 3.1. Manageable Disability

I consider myself to be very—you know, I'm very happy. And, you know, when I am not clear about something, I just talk to [husband] about it or figure it out myself. I mean, it takes me a little longer, perhaps, than somebody who did not have Alzheimer's, but, you know, that's not a problem. You're just sometimes a little bit slower. Wouldn't you say? [asking husband, who concurs] So, I mean, we sort of do things together (female, with AD).

We think that even now in most—if you get early diagnosis, it's a manageable disability if you get the help, you know, a little bit of help (male, wife with AD).

I do not want to focus on [Alzheimer's]. I do not need to. So far, we are going along fine. We are both happy and we sleep together. He tells me 5 or 10 times a day that he loves me. He gives me a kiss and hug when he goes down stairs and comes back. He thanks me for every meal. He washes all my dishes. He has for years and years. He does not do as many things around the house. But, he found an error in our statement that the bank had made that I overlooked. He's pretty sharp at that. He watches the bank statements (female, husband with AD).

Similar views were reported by all 10 dyads in our study. Diagnosed individuals' desire to avoid being equated with a given condition is well-established within the medical sociological literature [27, 28, 42, 62] and our findings demonstrate that the spouses of people with AD support them in this endeavor. Within relationships framed by positive experiences and a lifelong commitment [32], this shared outlook was the most common strategy to maintain “couplehood” [35, 36, 63] reported in our study. 

As suggested elsewhere [32], when the reaction to changes related to memory loss are experienced within the context of those associated with aging generally, they are perhaps less traumatic and/or disruptive. Furthermore, familiar environments with positive relationships and shared constructions of meaning support both couplehood and aging in place. Despite consensus on the importance of continuing to live as normally as possible and the positioning of memory loss as a “manageable disability”; however, the “united front” couples presented also involved within-dyad gender differences in coping strategies and disagreement over perceptions/outlook, as reported elsewhere [64].

### 3.2. Support Persons Find Individual “Tricks” versus Group Support

Female support persons were more likely than their male counterparts to rely on concrete strategies such as note writing and calendar keeping in helping them deal with their spouses' memory loss:

I have to find out the different tricks myself. We have a calendar on the refrigerator and I have to put everything on the calendar…where I will be at, if I have to work, if I'm getting a hair cut or getting my nails done, we're going out to dinner. Whatever we are doing I put on that calendar (husband with MCI).

I've always put notes around. I've tried to get [my husband] to do it but he wouldn't do it. He wouldn't put notes around. He was fine if you gave him a note and you left him a list (husband with MCI).

Male support persons, in contrast, commonly turned towards interactive, collective resources such as support groups or churches as a form of coping with their wives' memory loss.

We have a great group at church [that provides support] for everyone dealing with this (wife with AD).

There's one thing that I wanted to talk more about with the support group—one of my neighbors right down the street here has got a similar situation, and she goes to the support group, the same place I do, but at a different time. And I just wonder if maybe we could all get together and go to the same support group, which would be a little bit better (wife with AD).

Although spousal carers report similar efforts at “managing forgetting” [65] as do diagnosed individuals themselves [66, 67] male and female support persons in our study ultimately found different strategies helpful in coping with their spouses' memory loss. While our data perhaps corroborate suggestions of a gendered dynamic to caregiving, including increased burden for wives [68], husbands more likely to normalize symptoms [69], husbands having more outside support available to them and less restrictive social activities [66, 69, 70], and problem-solving (male) versus hands-on emotional (female) coping [71], it is crucial to acknowledge the social determinants of health and cultural influences on beliefs, especially in such a small study, to avoid reductionist claims based on gender. 

### 3.3. Support Persons Promote Autonomy versus Admitting Discontent

Although memory loss can greatly decrease someone's ability to perform certain tasks, our female support persons promoted as much autonomy as possible in their spouses, seen most clearly in the instances of driving and use of humor to minimize problems.

The time will come when we will have to talk about a driver's evaluation. But I do not think I'm going to be the one to talk about it, and that's what they wanted me to do…And I just—I'm no expert. I'm in no position to say “[Husband's name], you'd better have your driving evaluated” (husband with AD).

He used to forget directions. When we drove anywhere, very familiar places, he would forget how to get there. And when he got there, he would forget how to get home. If he went into a parking lot, he would not know which way to go back. Sometimes, I would just let him go and he would say, “what are we doing here?” I'd say, “I thought you were taking me out someplace new for dinner.” We have continually laughed. I think is very beneficial for us (husband with MCI).

Occasionally, he'll go down stairs to pick up the mail. He'll come back up and he does not have it. “Oh you do not have the mail.” “Oh, I forgot.” He'll go back down and do it. Sometimes and goes backwards like this and the blood goes out of his head or something. If there is something that could be taken as a reprimand or disappointment, like he forgot to bring the paper or he did not bring the mail, I've learned not to say anything. It's better to let it go (husband with AD).

While these data suggest that perhaps wives providing care are more concerned with maintaining reciprocity in relationships or active engagement with their husbands despite memory loss, it is equally plausible that it underscores the need to understand the “contextual nature” of husbands' narratives [72]. Likewise, these data may lend support to findings that relational aspects were more important for female than male carers [68], but they might also demonstrate the difficulty of challenging traditional gender roles for women [69] and/or caregiving wives' stronger concern with how other people perceive their husbands. Such “saving face” strategies have been reported elsewhere [66].

Unlike previous findings [35], however, our male support persons did not focus solely on meeting their wives needs or upholding their autonomy. In concert with other studies [56], our data reveal husbands who vocalized the discontent and disruption that they faced in providing care for their wives with Alzheimer's.

Well, a few times she's gone out and gotten lost, and I've gotten kind of worried about it…There's no place else that she goes [other than on walks] really. I have to let her do that. Everything else, I go with her, which is kind of a nuisance sometimes (wife with AD).

I've found it hard even to go out and cut the grass because if I'm out there an hour, she's probably out there 3 or 4 times asking me a question (wife with AD).

While the wives in this study stressed their husbands' independence and their retained capacity for making valuable contributions, the husbands providing care were more vocal about their own hardships. This focus on “other” versus “self” is a potentially important distinction in methods of caring, which demonstrates the inadequacy of supportive services that assume a uniform approach based on the perspectives of predominantly female carers.

### 3.4. Perceptions of AD According to Diagnosis

As reported elsewhere [64], there were also important differences between diagnosed individuals and their spouses as regards reactions to and interpretations of memory loss itself and perceptions of the future. The following quotes demonstrate this divergence in defining Alzheimer's.

It's an old disease. [It's] just the deterioration of your brain capacity (male, MCI).

[AD] isn't a disease. It's an animalistic type of thing I think because you can see it in a lot of animals as they get older. They don't act the same (male, AD).

In contrast, spouses reveal different interpretations.

It's a disease of your brain that incapacitates gradually and you become less of who you were. You lose who you were. You become less…you do not lose who you are (female, husband with MCI).

[Alzheimer's is] The loss of self as others know you, as others knew you (female, husband with AD).

While diagnosed individuals defined AD in vague terms, and MCI with even less clarity, their spouses used far more medicalized terminology for both. As other studies suggest [66], many spousal support persons reflect broader cultural views when they assume diagnosed individuals experience a “loss of self.” Consequently, carers envision the situation to be worse, or “harder,” for them than it is for their counterparts. For example, many participants shared sentiments along the lines of this woman: “It's harder for me to perceive than for him to experience from what I gather because he is so content” (husband with MCI).

In contrast, diagnosed individuals discuss a “gradual,” “normal,” and “manageable” decline of memory perceived to be associated with aging and do not report distress over being diagnosed. For example, the following men depict their experiences accordingly:

PI: Is the diagnosis important to you? Diagnosed Husband: No. Wife: He's not broken up about it. PI: Is that because you think it is normal or do you think it is a disease? Diagnosed Husband: I'd go along those former lines. I'll tell you, as you get older why you forget; you have more things to remember. You remember the important things and throw out the things that aren't important. PI: So, you're not overly concerned? Diagnosed Husband: No (male, AD).

If you are drinking and smoking, that affects your memory. If you are just lazy and don't think about anything, you'll forget. I like to read. I like to study. I like to meet people and walk around. Keep my actions going (male, AD).

PI: So you feel like things are going well for you? Yes, I have no regrets of anything. We try to get out and do something every day (male, AD).

These findings are consistent with recent studies suggesting an inverse relationship between privilege—as regards social location along axes such as race, class, and gender—and negative views of dementia [72]. Diagnosed individuals in this study experienced their losses as less severe than their spouses perceived them to be, tending to see their symptoms as either “not a big deal” or a “nuisance” rather than “hellish.” If diagnosed individuals fear being socially marginalized, then one might also expect them to minimize the significance of their condition to avoid being conflated with AD and resist the subsequent social disenfranchisement [26, 27, 72]. Our data support previous studies recommending that carers follow the lead of those diagnosed and provide help as needed rather than preemptively [73]. 

Closely related, significant discrepancies also existed between diagnosed individuals and their spouses about the perception of the future:

PI: How do you see your future playing out? Husband: Fantastic. It's going to be all right. If you aren't dead, you aren't anything. That's all. Sure. I think I've been lucky at it so far. Because if you consider the percentage that make 80, it's a very small percentage probably.

Wife: It's a challenge. It's a problem. You've got a problem, you do what you can and you do not give up and you adapt. It's up to me to make myself happy, nobody else. I find things that give me what I need. Our life together is a challenge (husband, MCI).

As discovered elsewhere [36, 46, 74], these different reactions and interpretations potentially lead couples to experience the effects of memory loss in ways that can be incompatible. As a result of the different reactions, interpretations, and experiences within couples, according to both gender and diagnosis, previously harmonious “joint lived experiences” [28] within spousal dyads may be threatened [46]. In the joint career of memory loss, most of our dyads—in contrast—remained couple-focused, representing Kaplan's [36] “Til death do us part” or “We—but…” views. Despite this finding, the potential “compromised mutuality” [39] does pose a possible risk to the very interconnectedness and compassion of couples “working together” [75], which is integral to both providing and receiving high-quality, relationship-centered care. If we believe the potential for “couplehood” [33, 35–37, 63] to be unifying rather than dichotomizing between the personhood of each spouse separately, then it is important to understand these discrepancies and foster open dialogue and connection among the most intimate of social groups. 

The only longitudinal ethnographic study of persons with Alzheimer's and their families that we are aware of presents strong evidence that caring relationships are “dynamic co-constructions built upon everyday events, interactions, environments, and disease progression” [59, page 335]. Accordingly, cooperative care relationships are built on foundations of mutual respect and sensitivity to persons with dementia whereas lack of trust and compassion leads to negative outcomes based on unrealistic expectations and retaliation efforts. If spousal carers do not perceive the work they are doing as “caregiving” but rather as an extension of their relational role, then a potential unintended consequence of the caregiver designation is to discredit the person with memory loss [76]. Our cross-sectional data support both the “co-constructive” nature of caring and the perception of so-called caregiving as an extension of their existing relationship rather than a new role, thus cultivating positive adaptations to memory-related changes and barriers to in-home living for both parties. These data highlight the power of couplehood and significance of relationship status. It is important to account for potential costs/benefits of marital relationships since while we found a protective role of marital status here, relationships could instead pose a threat. 

## 4. Discussion

To better understand the impact memory loss has on aging in place vis-à-vis intimate relationships, this article examined the efforts to maintain personhood and couplehood within spousal dyads confronted with AD or MCI. Informed by symbolic interactionism and social constructionism, data suggest that experiences of memory loss entail not only the perspectives of each spouse but also their shared relationship or “marital biography” [77]. By investigating the living-in-relationships of our dyads [77] and context awareness theory [35] in the “dynamics of dementia” [75], our data reflect the tremendous investment made by couples to preserve a working (“together”) relationship. Our results reveal the complexity of dementia as something that is *shared*, for better or for worse, by offering an intimate understanding of the lived experiences of spousal dyads. 

Our data support the conclusions of those who argue that in the “joint career” of Alzheimer's, couples exert considerable effort to construct a “shared awareness” [31], creating opportunities for diagnosed individuals and spouses alike to remain active in their relationship together [28, 63, 75] rather than being a one-dimensional, negative experience for both parties as previous studies and common social (mis)conceptions purport. These findings also suggest, however, that couples do not always share completely consistent perceptions of memory loss. Nonetheless, we contend that rather than being “disrupted” or “transformed” [46] in a pejorative or definitive sense, most couples in our study showed clear indications of “working separately” or “working apart” [75], or a “compromised mutuality” [39] that vacillates over time. Our data demonstrate that in some relationships, changes were quite purposeful since interaction requiring direct recall and/or shared memories or coping strategies grounded in interactional similitude may no longer benefit the relationship or be feasible. While we do not wish to downplay the fact that many couples ultimately find themselves “working separately” or “working apart,” we acknowledge that these stages are not universal, linear, or entirely fraught and dismal. Contrary to Keady and Nolan's [75] argument that “working together” is the “best case scenario,” the idea that “working” itself is couple-specific fits more closely with our data. Couples, either jointly or independently, managed to configure livable arrangements (i.e., find an “emergent fit”) [78, 79] that do not always reflect congruence or shared worldviews, but there was a common commitment to “doing things together” [35] that disputes conceptions of “caregiving” or living with Alzheimer's in an exclusively disparaging or isolating light. 

Although these data may represent one of the social (and personal) benefits of spousal relationships for aging in place; that is, positive dyad relationships and familiar environments, or “physical insideness” [6], are perhaps protective against nursing home placement, it is important to highlight that there is a continuum of experience throughout this pendular process. Given that our respondents were all in the early stages of memory loss, their experiences are likely to change over time. Since awareness context theory is based on relational capacity and therefore negotiated [35], couples may go through any one component of awareness at a given period in their relationship and revisit phases as needed. Whereas “covering one's tracks” [75] would mean that one member of a couple is in *suspended open awareness *and beginning to isolate, nowhere in our sample did we find couples in denial of the diagnosis, let alone in a relationship they cast as fraught or overwhelming. In contrast, couples that included one member who ignored, downplayed, or outright rejected the “master status” of diagnosis understood this as a means of coping and logical extension of enjoying life to the fullest rather than pathologizing it as “denial.” As has been reported elsewhere in the literature [28, 35, 58], individuals diagnosed with dementia (are forced to) work especially hard to avoid being conflated with their disease, a predictable consequence of a highly medicalized culture [27]. Our data demonstrate that their spouses work equally hard to support them in this.

The efforts to downplay AD/MCI reinforce recent discoveries that couples resist information on the disease and instead focus their energies toward positive thinking [35]. The humor and continued efforts at socialization, among other methods, reported by our respondents corroborate these findings. Several couples reported feeling at ease with so-called “symptoms” of memory loss and resultant life changes. Others (re)framed information-seeking as an attempt to achieve closure, confidence, and control over a disease that initially appeared foreign, overwhelming, and strictly negative. Engaging in such illness work is again highly couple-specific, with each pair (and perhaps individual within it) reacting differently. Accordingly, partners do not need to react the same way to be coping “well” as a couple. As reported elsewhere [72], the idiosyncratic nature of couplehood with AD means that a universal experience of the condition does not exist. Instead, the various experiences reflect the relative social positioning, or age, racial, ethnic, and class background, of the couples involved, here married, well-educated, middle-class, Caucasian seniors.

Our data also engage debates on dementia and the “self.” Existing studies posit that support persons engage in the bulk of restorative identity work *on behalf of those diagnosed *by reframing troubling symptoms as normal occurrences, downplaying their significance, or avoiding situations that would expose their loved ones' shortcomings (or encouraging diagnosed partners to do so). The present data dispute this as the majority of our spouses proved highly adaptive to changes brought on by memory loss and negotiated meaning jointly with their diagnosed spouses. For example, in contrast to arguments that those with memory loss are unable to restore a familiar self [46], our findings show quite clearly that persons in the early stages of AD/MCI, too, are savvy, strikingly resilient, prove equally adaptive as their spouses, are decision makers, and are opinionated. Rather than a loss of personal identity, our data suggest that as a result of the medicalized worldview dominant in contemporary society, it is the social self that is threatened by a diagnosis of memory loss. Our respondents further demonstrate (the need for) joint efforts to combat such social relegation [27]. This may suggest a limitation in our sample of relatively “privileged” people, both in terms of social location and severity of memory loss, who are afforded the luxury of addressing the “socioemotional” aspects of the condition rather than worrying about the difficulties in performing everyday instrumental tasks such as cooking, paying bills, and shopping that often accompany the condition [72] (While this may be due to a lack of diversity (i.e., marginalization) in the sample, the respondents are arguably underprivileged according to diagnosis and age. This interesting line of inquiry merits further elaboration but is beyond the scope of the present paper.). 

In an effort to combat the “absence of self” rhetoric, many couples in our sample continued to maintain familiar social or recreational activities, or “social insideness” [6]. These interactions with the social world were meaningful and benefited persons with memory loss and their spouses, just as they would anyone else. By reconnecting couples with familiar, joint activities [53], appreciation for each other and ultimately “couplehood” can be fostered. While, of course, no amount of identity work can erase the biological effects of Alzheimer's, our data demonstrate that a couple's restorative strategies do much more than manage the other's presentation of self [45]. That is, the paradox of working toward congruence in a relationship where one member is deteriorating does not stop couples from creating comfortable living conditions for each other and, in fact, in some instances it even engenders couplehood. Indeed, life with AD/MCI may shift spousal roles, but core elements of the identities of both individuals and the dyads they inhabit can be preserved and even strengthened. 

Since meanings are socially constructed, the influence of memory loss will be different for every couple [32] and the joint production of meaning should be expected to shift over time. Through listening to the common threads among each couple's unique story, the social bond of coupling can be better understood. In particular, our data support the belief that couples dealing with dementia together are more likely to remain positive, maintain a sense of self and couplehood, and evade the typically negative framework attached to dementia in America than might be the case for either party individually or single/widowed seniors generally. As reported elsewhere, spouses (can) play a crucial role in enabling persons with memory loss to remain at home [77]. Our findings thus challenge representations of memory loss as an exclusively negative and isolating experience and support existing research [56, 65] by demonstrating the strength of spousal bonds in helping both diagnosed individuals and their counterparts age well, maintain dignity and value, and remain in their homes for as long as possible. As others have argued [9], understanding how efforts to remain at home can support the preservation of self for persons with dementia warrants further study, including longitudinal analysis.

## Figures and Tables

**Table 1:** Study demographics.

	Mr.* and Mrs. J	Mr.* and Mrs. D	Mr.* and Mrs. R	Mr.* and Mrs. F	Mr. and Mrs. M*	Mr. and Mrs. A*	Mr. and Mrs. B*	Mr. and Mrs. K*	Mr.* and Mrs. S	Mr.* and Mrs. P
*Age*										
45–55 years			X X							X X
56–64 years									X X	
65–74 years	X			X X				X		
75–84 years	X				X X	X X	X X	X		
85+ years		X X								
*Race/ethnicity*										
African American										
White, not Hispanic	X	X	X	X	X	X	X	X		X
Hispanic or Latino/a									X	
*Marital status*										
Married	X	X	X	X	X	X	X	X	X	X
*Living status*										
Coreside	X	X	X	X	X	X	X	X	X	X
*Residence*										
Rural					X					
Urban	X	X	X	X		X	X	X	X	X
*Education status*										
< High school										
High school graduate or GED									X	
Some college, technical/vocational		X	X		X X					
≥ College degree	X	X	X	X X			X X	X X		X X
*Annual income*										
<$20,000									X	
$20,000–$39,999										
$40,000–$64,999					X		X			
$65,000–$99,999	X	X						X		X
>$100,000			X	X		X				
*Residence*										
Rural					X					
Urban	X	X	X	X		X	X	X	X	X
*Diagnosis*										
AD		X			X	X		X		
EOAD			X						X	X
MCI	X			X			X			
*Time since diagnosis*										
2-3 years	X			X	X					
3-4 years		X	X			X	X		X	X
5+ years								X		

*This individual was the spouse who had been diagnosed with AD/MCI.

**Table 2:** Tentative interview guide.

(A) Subjective experience	
(i) When did you first realize changes in X's memory?	
(ii) Who noticed the changes first? Did you feel comfortable sharing your thoughts with the other?	
(iii) Tell me about your experiences leading up to being diagnosed.	
(iv) How do you define Alzheimer's disease (AD)/mild cognitive impairment (MCI)?	
(v) What does AD/MCI mean to you?	
(vi) What has your experience been like since the diagnosis?	
(vii) What are the biggest changes that have happened in your life since the diagnosis?	
(viii) How would you describe your relationship prior to diagnosis?	
(ix) How, if at all, has your relationship changed since the diagnosis?	

(B) Social experience	

(i) How, if at all, have your interactions with others changed since diagnosis?	
(ii) Tell me how you think your diagnosis affects your loved ones, if at all.	

(C) Miscellaneous	

(i) Are there any unexpected things that have come from your experiences with Alzheimer's? Any “silver lining” to your situation?	
(ii) How do you envision the future?	
